# Association of Serum Bilirubin with the Severity and Outcomes of Intracerebral Hemorrhages

**DOI:** 10.3390/antiox10091346

**Published:** 2021-08-25

**Authors:** Kai Fu, Cynthia S. Garvan, Shelley C. Heaton, Nandakumar Nagaraja, Sylvain Doré

**Affiliations:** 1 Department of Anesthesiology, University of Florida College of Medicine, Gainesville, FL 32610, USA; kaikaimit@ufl.edu (K.F.); cgarvan@anest.ufl.edu (C.S.G.); 2 Department of Clinical & Health Psychology, University of Florida College of Public Health and Health Professions, Gainesville, FL 32610, USA; sheaton@phhp.ufl.edu; 3 Department of Neurology, University of Florida College of Medicine, Gainesville, FL 32610, USA; Nandakumar.Nagaraja@neurology.ufl.edu; 4 Center for Translational Research in Neurodegenerative Disease, Departments of Psychiatry, Pharmaceutics, and Neuroscience, McKnight Brain Institute, University of Florida College of Medicine, Gainesville, FL 32610, USA

**Keywords:** albumin, free bilirubin, hemoglobin, intracranial bleeding, stroke

## Abstract

Intracerebral hemorrhage (ICH) is the second most common subtype of stroke, and it is often associated with a high mortality rate and significant morbidity among survivors. Recent studies have shown that bilirubin, a product of heme metabolism, can exhibit cytoprotective, antioxidant and, anti-inflammatory properties. However, little is known about the role of bilirubin in combating several pathophysiological pathways caused by intracerebral bleeding in patients with ICH. In this study, data were collected retrospectively on 276 patients with ICH who were admitted to a university hospital between 5 January 2014 and 31 December 2017. We assessed the relationship between levels of total, direct, and indirect serum bilirubin and assessments of initial stroke severity and clinical outcomes by using Spearman’s rank correlation and Kruskal-Wallis H tests. A secondary examination of the carrier protein albumin was also undertaken. Our study found that higher levels of direct bilirubin were correlated with worse admission Glasgow Coma Scales (GCS) (*r_s_* = −0.17, *p =* 0.011), worse admission ICH Scores (*r_s_* = 0.19, *p* = 0.008), and worse discharge modified Rankin Scales (mRS) (*r_s_* = 0.15, *p* = 0.045). Direct bilirubin was still significantly correlated with discharge mRS after adjusting for temperature at admission (*r_s_ =* 0.16, *p =* 0.047), oxygen saturation at admission (*r_s_* = 0.15, *p* = 0.048), white blood cell count (*r_s_* = 0.18, *p* = 0.023), or Troponin T (*r_s_* = 0.25, *p* = 0.001) using partial Spearman’s correlation. No statistical significance was found between levels of total or indirect bilirubin and assessments of stroke severity and outcomes. In contrast, higher levels of albumin were correlated with better admission GCS (*r_s_* = 0.13, *p* = 0.027), discharge GCS (*r_s_* = 0.15, *p* = 0.013), and discharge mRS (*r_s_* = −0.16, *p =* 0.023). We found that levels of total bilirubin, direct bilirubin, and albumin were all significantly related to discharge outcomes classified by discharge destinations (*p* = 0.036, *p* = 0.014, *p* = 0.016, respectively; Kruskal-Wallis H tests). In conclusion, higher direct bilirubin levels were associated with greater stroke severity at presentation and worse outcomes at discharge among patients with ICH. Higher levels of albumin were associated with lower stroke severity and better clinical outcomes. Future prospective studies on the free bioactive bilirubin are needed to better understand the intricate relationships between bilirubin and ICH.

## 1. Introduction

Stroke is the fifth leading cause of death in the United States, affecting nearly 800,000 Americans each year [1]. An epidemiology study found that the incidence of stroke per 100,000 population was approximately 218 in men and 127 in women [2]. Intracerebral hemorrhage (ICH) is the second most common type of stroke, constituting 10% to 15% of all stroke cases in the United States, and it is associated with a high case fatality rate of up to 40% at 30 days [3]. ICH occurs when a blood vessel ruptures inside the brain, leaking toxic blood components to the surrounding tissues and causing irreversible damage. The extravasated blood can collect and grow into an expanding hematoma that increases intracranial pressure and causes mechanical compression of other brain structures. Secondary brain injuries from the hemorrhage can be attributed to several parallel physiologic responses to the cytotoxic, oxidative, and inflammatory effects of blood products and hematoma [4,5,6,7,8]. Although trials for new ICH treatments have grown significantly in recent years, few surgical or pharmacologic therapies have been demonstrated to be effective in reducing mortality or improving outcomes among survivors [9]. The lack of established treatments for this devastating disease makes it necessary to search for novel therapies that target the pathophysiological mechanisms of ICH.

Bilirubin is a yellow, lipophilic, linear tetrapyrrole compound generated by the catabolism of heme, a component mostly derived from hemoglobin in red blood cells. In the heme catabolism pathway, heme is first cleaved by heme oxygenase enzymes to produce biliverdin, which is reduced to unconjugated bilirubin by biliverdin reductase. Because the unconjugated bilirubin is toxic and insoluble, it must be bound to albumin, a transport protein in plasma, before being delivered to the liver. Once reaching the liver, unconjugated (or indirect) bilirubin undergoes conjugation with glucuronic acid, a step that is catalyzed by glucuronosyltransferase. The conjugated (or direct) bilirubin is subsequently excreted to the intestines as part of bile [10]. In the past, bilirubin was generally considered to have few physiological functions and even toxic if it accumulated in the human body. However, recent studies have shown that bilirubin also exhibits antioxidant, anti-inflammatory, and cytoprotective properties [11,12,13,14,15]. When acting as an antioxidant, bilirubin can reduce reactive oxygen species, and it is consequently oxidized back to biliverdin. If the level of biliverdin reductase is high enough, biliverdin can be reduced to regenerate bilirubin. This bilirubin-biliverdin redox cycle might explain its ability to reduce oxidative stress, inflammation, and cytotoxicity even when it is present in low physiologic concentrations [15,16,17]. These therapeutic properties are especially important for organs that do not have strong endogenous cytoprotective defenses. For example, there is evidence that bilirubin can protect neurons against oxidative stress injuries, which commonly occur after ICH [12].

A small fraction of bilirubin circulates as free, unbound, and unconjugated bilirubin. The fraction of free bilirubin increases when the concentration of unconjugated bilirubin exceeds the binding capacity of albumin or when the binding affinity for bilirubin is interfered with by certain medical conditions or the presence of inhibitors that compete with bilirubin for albumin binding [10]. Due to its small size, hydrophobicity, and amphipathic nature, free bilirubin can freely cross the intact blood-brain barrier and penetrate neuronal membranes. There is strong evidence that free bilirubin concentration, rather than total bilirubin or unconjugated bilirubin, determines bilirubin distribution and the risk of neurotoxicity induced by hyperbilirubinemia [10]. However, routine clinical chemistry analyzers in the United States, unfortunately, do not determine free bilirubin, and no widely available method that rapidly and accurately measures the concentration of free bilirubin exists. Current FDA-approved methods, such as Zone Fluidics (a peroxidase method) and UB Analyzer (a modified peroxidase method), have technical and practical limitations that prevent them from being used routinely; therefore, they are confined to only specialized clinical laboratories [18,19,20,21]. Some studies have shown that total bilirubin or the ratio of total bilirubin to albumin could be used to estimate free bilirubin [22,23,24]. 

Existing clinical studies on the relationship between bilirubin and stroke yield conflicting and mixed results [25,26,27,28,29,30]. Overall, studies generally show that higher serum bilirubin levels are correlated with reduced stroke risk but increased stroke severity and worse clinical outcomes for ischemic stroke. The association between serum bilirubin and hemorrhagic stroke remains unclear. In addition, many studies did not differentiate between different types of bilirubin (direct, indirect, and total). Some studies only considered either the stroke severity or clinical outcome but not both. Therefore, this retrospective study was designed to investigate the association between different forms of bilirubin (direct, indirect, and total) with the initial stroke severity at admission and clinical outcomes at discharge. Because of the close association between bilirubin and its carrier protein albumin, we also explored the relationship between albumin and ICH. Additionally, we investigated the correlation of different forms of bilirubin and albumin with factors known to be associated with ICH outcomes (e.g., age, blood pressure, and laboratory measurements).

## 2. Materials and Methods

### 2.1. Human Subject Selection Criteria

This retrospective study included 276 patients with ICH who were 18 and older and admitted to UF Health Shands Hospital for ICH between 5 January 2014 and 28 December 2017. The University of Florida Institutional Review Board approved the study. All subjects in this study had available data on total and/or direct bilirubin levels, which were collected within a week of admission and recorded in a standardized fashion in their electronic medical records. Study exclusion criteria included pregnancy, current cocaine or amphetamine use, sepsis, and medications known to alter bilirubin levels (see Table S1). Patients with intraventricular hemorrhage and patients who suffered hemorrhagic transformation from a primary ischemic stroke were also excluded from our study. A total of 697 patients were admitted to UF Health Shands Hospital for ICH between 5 January 2014 and 28 December 2017. Available medical records of 519 patients were thoroughly reviewed. Of the 519 patients, 333 patients had available data on total bilirubin and/or direct bilirubin after admission, and 276 patients met the exclusion criteria mentioned above. 

### 2.2. Data Extraction

Three trained investigators extracted data from the electronic medical record. General health and demographic characteristics including vital signs, social history, and past medical history were recorded. In addition, laboratory measurements, taken mostly within 7 days of admission, such as the liver function test, complete blood count, and comprehensive metabolic panel, were included.

The initial stroke severity was assessed using Glasgow Coma Scale (GCS) and ICH scores that were recorded at hospital admission. The GCS is a clinician-assigned rating scale intended to assess the level of consciousness based on patient behaviors observed in the domains of eye opening, motor response, and verbal response. The GCS generates a total score with a minimum value of 3 and a maximum of 15, with higher scores indicating better neurological status. The ICH Score is a scale used by physicians to clinically grade the medical severity of patients presenting with ICH and gauge the associated risk of mortality. The score is calculated by assigning ICH Score points across four prognostic indicators: GCS upon admission, patient age, ICH volume, and lesion location. The ICH Score generates a total score with a minimum value of 0 and a maximum value of 6, with worse ICH scores associated with higher mortality risk.

Clinical outcomes were recorded across several indices: mortality, length of hospital stays, GCS at discharge, modified Rankin Scales (mRS) at discharge, and discharge destinations. Given the rate of missing data for discharge GCS and mRS scores, mortality and discharge destination information were used to classify patients into either “positive,” “negative,” or “death” outcome categories. This was used as the primary measurement of clinical outcomes. More specifically, patients discharged to home or acute inpatient rehabilitation facilities were considered to have a good or “positive” clinical outcome. In contrast, patients discharged to long-term care facilities, skilled nursing homes, subacute rehabilitation facilities, or hospice were considered to have a poor or “negative” clinical outcome. GCS and mRS scores at discharge were also examined and considered when classifying the discharge outcome. The discharge GCS follows the same description as the admission GSC score (see above). The mRS is used by physicians to measure the degree of disability or dependence in daily activities at discharge. Scores range from 0 (no disability) to 6 (death), with higher scores indicating worse functional outcomes at discharge.

### 2.3. Statistical Analysis

Statistical analyses in this study were performed using SAS version 9.4 (SAS Inc., Cary, NC, USA) software. The levels of indirect bilirubin were calculated by subtracting direct bilirubin from total bilirubin. Data were first inspected for missing values and distributional forms. Descriptive data were determined for all variables of interest. The associations between total, direct, and indirect bilirubin and various assessments of initial severity and clinical outcomes were analyzed using Spearman’s correlation. The levels of bilirubin were compared among patients with positive, negative, and death outcomes using Kruskal-Wallis H tests. To identify possible confounding variables, relationships between discharge mRS and candidate variables including medical conditions, vital signs, albumin, and other laboratory measurements were analyzed by using Spearman’s correlation (for ordinal and continuous variables) and Wilcoxon rank-sum tests (for binary variables). Analyses were then conducted to investigate the association between bilirubin and discharge mRS after adjusting for significant confounding variables using partial Spearman’s correlation testing. All testing was two-tailed, with a level of significance of 0.05. A sample size of 276 allows for the detection of a correlation of 0.17 or higher in magnitude.

## 3. Results

A total of 276 patients met the study inclusion and exclusion criteria. The average age of patients was 65.8 ± 16.2 years. One hundred forty-eight (54.6%) were males and 128 (46.4%) were females. General characteristics of patients, including medical history and social history, are shown in Table 1. Hypertension was the most common pre-existing medical condition, present in 184 (70.2%) patients. Fifty-six (21%) and 49 (18.7%) patients had diabetes and hyperlipidemia, respectively. Around half of the patients (87, 52.1%) smoked cigarettes or had smoked cigarettes in the past. A little more than a third of the patients (68, 39.5%) drank alcohol either socially or regularly. 

Initial evaluations, including vital signs, GCS scores, and ICH Scores at admission, and primary hemorrhage locations are shown in Table 2. The mean blood pressure was 155/83 mmHg. Eighty-three patients (30.2%) were already at a GCS score of 8 or lower at admission. The most common lesion location was lobar structures in 111 (40.2%) patients. Lobar structures were the most common location where bleeding takes place. A total of 208 (75.4%) patients did not receive any surgical treatments besides the diagnostic angiography.

Outcome measurements, including lengths of stay, in-hospital deaths, GCS and mRS scores at discharge, and discharge destinations are shown in Table 3. The mean length of hospital stay was 8.7 days. Sixty-one (22.1%) patients died during their hospital stay, and another 22 (8.0%) patients were discharged to either a hospice facility or home with hospice services. A total of 81 (30.0%) patients were at a GCS score of 8 or less at discharge. Overall, less than a third of the patients (29.0%) were considered to have positive discharge outcomes, and 136 (48.9%) patients were considered to have negative discharge outcomes based on our discharge location classification, described in the previous section.

Descriptive data regarding levels of serum bilirubin, albumin, and other laboratory measurements are presented in Table S2. The mean values of serum total, direct, and indirect serum bilirubin and albumin were 0.65 mg/dL, 0.51 mg/dL, 0.16 mg/dL, and 3.72 g/dL, respectively. As a reference, the normal range is 0.1 to 1.2 mg/dL for total bilirubin, <0.3 mg/dL for direct bilirubin, and 3.5 to 5.5 g/dL for albumin. Correlations between different forms of bilirubin or albumin and other clinical and laboratory factors known to be associated with ICH outcomes (e.g., age and blood pressure) are displayed in Table S3. 

**Figure 1:**
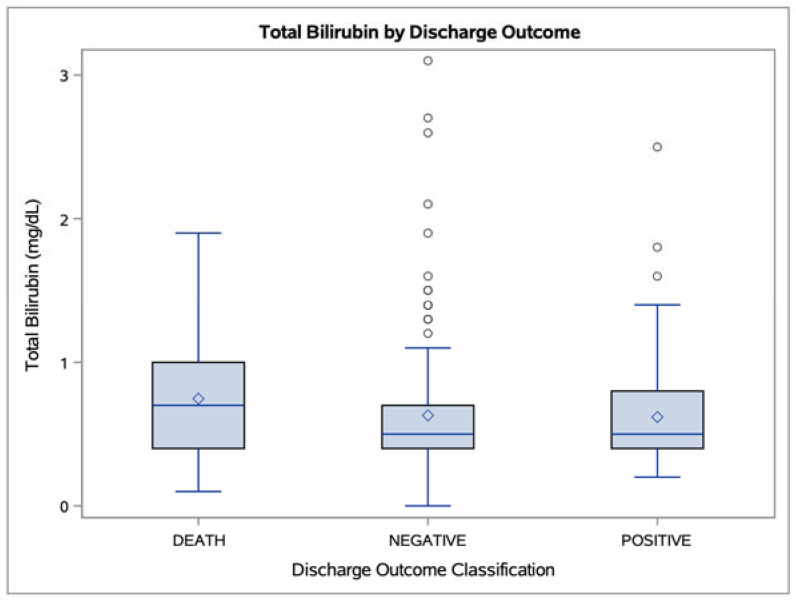
Boxplots of total bilirubin levels observed in “Death” (n = 61), “Negative” (n = 136), and “Positive” (n = 80) discharge outcome groups (*p* = 0.036). Mean values are represented as white diamonds; outliers are represented as white circles.

**Figure 2:**
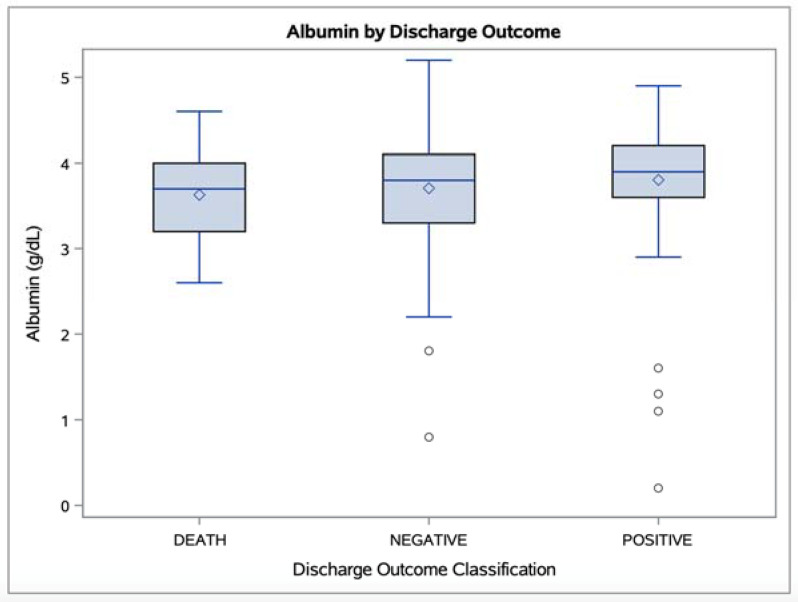
Boxplots of albumin levels observed in “Death” (n = 61), “Negative” (n = 136), and “Positive” (n = 80) discharge outcome groups (*p* = 0.016). Mean values are represented as white diamonds; outliers are represented as white circles.

Compared to patients who died or who had other negative outcomes (e.g., hospice), patients with better outcomes had lower levels of total bilirubin (*p* = 0.036) and direct bilirubin (*p* = 0.014) but higher levels of the bilirubin transport protein albumin (*p* = 0.016). The distributions of total serum bilirubin and albumin levels observed in the different outcome subgroups are depicted in Figure 1 and Figure 2. 

According to the Spearman’s correlation results in Table 4, direct bilirubin was correlated with the GCS at admission (*r_s_* = −0.17, *p* = 0.011), admission ICH score (*r_s_* = 0.19, *p* = 0.008) and discharge mRS (*r_s_ =* 0.15, *p* = 0.045). No statistical significance was found in the correlation between levels of total or indirect bilirubin and assessments of stroke severity and outcomes. Serum albumin was found to be positively correlated with discharge GCS (*r_s_ =* 0.13, *p* = 0.02) and negatively correlated with discharge mRS (*r_s_* = −0.16, *p* = 0.023). 

We ran bivariate analyses (Wilcoxon rank-sum tests for binary variables and Spearman’s correlation tests for ordinal and continuous variables) for variables of interest that are potential predictors of discharge mRS (see results in Table S4). Subsequently, we included variables that were significantly correlated with discharge mRS (*p* < 0.05) in partial Spearman’s correlation tests, and we found that direct bilirubin was only correlated with discharge mRS after adjusting for temperature at admission (*r_s =_* 0.16, *p =* 0.047), oxygen saturation at admission (*r_s_* = 0.15, *p* = 0.048), white blood cell count (*r_s_* = 0.18, *p* = 0.023), or Troponin T (*r_s_* = 0.25, *p* = 0.001). 

## 4. Discussion

Our results suggest that higher direct bilirubin levels are associated with greater initial stroke severities and worse clinical outcomes in patients with ICH. Overall, patients with higher direct bilirubin levels at admission had worse neurological statuses and poorer prognoses upon admission (as evidenced by worse admission GCS and ICH scores). Patients with higher direct bilirubin also had greater levels of post-stroke disability at discharge, as evidenced by worse discharge mRS. These relationships could suggest that direct bilirubin could be a marker of oxidative stress in ICH. There are only a few existing clinical studies that examined the relationship between bilirubin and brain hemorrhages. A 2003 study on 23 patients with ICH found that serum bilirubin levels significantly increased in the early phases after the stroke onset [31]. One meta-analysis of five studies in the Chinese population found that patients with acute cerebral hemorrhage had elevated serum bilirubin [32]. A recent study by Xu et al. found that direct bilirubin was positively correlated with the mortality of patients with traumatic brain injury [33]. The authors of all three studies posited that higher serum bilirubin levels might have reflected the initial intensity of oxidative stress. Though, it is still possible that the level of protection conferred by bilirubin increased as more bilirubin was produced to meet the increased oxidative demand after a hemorrhagic stroke.

We found that total and indirect bilirubin was not associated with either stroke severity or clinical outcomes. It is unclear why total bilirubin did not show significant associations, but direct bilirubin did. This difference warrants future investigation with a larger patient population. A study on bilirubin and ischemic stroke yielded similar results; the authors found an independent relationship between stroke severity and direct bilirubin, but not with total bilirubin [30]. The authors reported that direct bilirubin may have better prognostic values than total bilirubin. However, previous studies also reported that total bilirubin or the ratio of total bilirubin to albumin could predict the level of free bilirubin and the neurological dysfunction caused by hyperbilirubinemia [22,23,24]. More work will be necessary to verify the hypothesis that direct bilirubin is a better prognostic factor. We also found that higher serum albumin levels were generally associated with more favorable outcomes, in contrast to bilirubin. Patients with higher albumin levels were more conscious at discharge and suffered less from disability or dependence, as indicated by the negative relationship between albumin and mRS. We found only few articles on albumin and hemorrhagic stroke. Our findings are supported by a study of 2010 ICH patients, which found that low serum albumin at admission is associated with lower admission GCS, higher mortality rates, and poorer functional outcomes [34]. However, numerous clinical studies on albumin and ischemic stroke overwhelmingly suggest that higher albumin levels are associated with better outcomes in ischemic stroke patients [35,36,37,38]. Albumin’s neuroprotective properties could be attributed to its antioxidant functions, hemodilution effects, ability to reduce edema, and modulation of immune responses [39,40]. Experimental studies suggest that human albumin therapy can improve neurological conditions following acute cerebral ischemia, but a randomized clinical trial did not find high-dose albumin therapy to have clinical benefits for patients with ischemic stroke [41,42,43]. 

Serum direct bilirubin levels were correlated with several vital signs and laboratory measurements. We found that higher serum bilirubin was generally associated with lower systolic and/or diastolic blood pressures. However, patients in our study were generally hypertensive at admission, as indicated by the elevated mean blood pressure in Table S2. A clinical trial found that reducing blood pressure after ICH onset resulted in better functional outcomes [9,44]. On the other hand, hypotension can also worsen clinical outcomes due to resulting acute cerebral vasospasm or cerebral ischemia after ICH [45,46]. Higher serum direct bilirubin levels were correlated with lower platelet counts, which might indicate increased platelet consumption and severity of bleeding. However, impaired platelet functions in patients could also have led to an increased propensity and severity of ICH. Finally, we found a significant positive correlation between serum bilirubin and liver enzymes ALT and AST. Previous studies have found that elevated liver enzymes are correlated with increased risk of ICH and worse clinical outcomes [47,48,49]. The exact mechanism is unclear, but alcohol consumption, hepatotoxic medications, and abnormal hemostasis may partially contribute to the adverse effects of liver dysfunction on ICH. Like other studies, we are reporting these correlations (see Table S3), and more work is needed to understand the physiological mechanism and clinical significance of these correlations.

There are several limitations associated with our study. First, the high number of statistical tests conducted increases the Type I error rate and effect sizes observed in key analyses were relatively small. Thus, the findings should be interpreted with caution and warrant reproduction in a larger sample size. Second, because this was a retrospective study, data were collected as documented in the electronic medical record, and some patients did not have all of the data points needed. In one study, a subgroup of patients with ICH determined to be lacunar hemorrhagic stroke likely due to hypertension had a favorable prognosis compared to non-lacunar hemorrhagic stroke [50]. Because our study was limited to retrospective chart review, we did not review the CT images to determine if patients had a hemorrhagic lacunar stroke. Third, most of the bilirubin data used in our study were collected within 3 days after admission with a few exceptions that were collected within 7 days. Thus, the limitation is that the first bilirubin value collected after admission may be different from the actual initial value, and various factors such as medical treatments could have interfered with the measurement. In future prospective studies, liver function tests should be consistently obtained within a few hours of admission to minimize such potential variabilities. Fourth, and most importantly, we did not have free bilirubin measurements in the patient data because the specific assay was not originally requested. Because free bilirubin is more bioactive and can cross the blood-brain barrier, free bilirubin is more likely to be responsible for bilirubin’s protective effects found in past studies [51]. Furthermore, the baseline functional status before ICH was also not documented, and data on the long-term functional outcome (after discharge) were not available. The lack of baseline functions could be a potential confounding factor when evaluating outcomes, especially if patients already had poor baseline functions before the onset of ICH. Finally, our study was limited in its outcome measurement as data were constrained to clinical outcome tools typically used in hospital settings that have lower measurement precision. Future studies with larger sample sizes and improved outcome precision measurements across different timepoints may allow for a better understanding of the relationship between bilirubin levels and the broad range of outcomes after ICH. 

Some studies have attempted to estimate free bilirubin through the ratio of total bilirubin to albumin. However, this is potentially problematic and does not improve total bilirubin prediction alone because there is a sizeable albumin polymorphism in the human population [43]. We considered several other ways to model or calculate bilirubin. For example, Ahlfors et al. modeled free bilirubin binding through a Henderson-Hasselbalch approach [52]. The bilirubin-albumin equilibrium was first simplified to Equation (1), where B_free_ represents free bilirubin, BBC represents bilirubin binding constant, B_total_ represents total bilirubin, and K_D_ represents the bilirubin-albumin equilibrium dissociation constant. Subsequently, the mass action equation and Henderson-Hasselbalch equation were used to derive the formula to calculate free bilirubin; see Equation (2). They measured the B_free_ and B_total_ of newborns by using the peroxidase method, and they calculated K_D_ by using Equation (3), an equation derived from Equation (2) [51]. After multiple calculations, they generated a table to show the relationship between K_D_ and levels of B_free_ and B_total_. However, our study included only adult patients who were over the age of 18, so it would be incorrect to assume that adults have the same dissociation constant as newborns because the albumin binding constants can change with age and vary between individuals. It would be optimal for future studies to include data on free bilirubin that are measured directly from the patients rather than calculated or estimated using a generalized formula.

(1) $$B_{\mathrm{free}}+\text{BBC}-B_{\mathrm{total}}\;\;K_{D}↔B_{\mathrm{total}}$$

(2) $$B_{\mathrm{free}}\;≅\;\frac{K_{D}\times \;B_{\mathrm{total}}}{\text{BBC}-B_{\mathrm{total}}}$$

(3) $$K_{D}\;≅\;\frac{B_{\mathrm{free}}\;\times \;\left(\text{BBC}-B_{\mathrm{total}}\right)}{B_{\mathrm{total}}}$$

In terms of future directions, a prospective clinical study with a larger sample size from multiple centers, blood samples collected immediately after admission, available free bilirubin measurement, and use of precision measurements of functional outcome collected prospectively up to 6 months after discharge are needed to better understand the relationship between bilirubin and ICH. Prospective clinical studies investigating the relationships between bilirubin and other forms of stroke, such as ischemic stroke and subarachnoid hemorrhage, are also required to distinguish further the effects of free and bioactive bilirubin in different oxidative environments.

## 5. Conclusions

The present work suggests that direct bilirubin levels are associated with greater initial stroke severity and worse clinical outcomes for patients with ICH. Total and indirect bilirubin were not associated with either stroke severity or clinical outcomes. Besides bilirubin, we also examined the association of serum albumin with ICH; higher levels of albumin were correlated with better clinical outcomes. Larger, controlled, multicenter prospective studies with available analytical data on free bilirubin, baseline function, and longer follow-ups are needed to better illustrate and understand the bilirubin’s potential prognostic value, beneficial effects, and biological significance.

## Figures and Tables

**Table 1:** Demographic and clinical characteristics of participants ^1^.

Variable	Mean (SD) or n (%)
Age (years)	65.8 (16.2)
Sex	
Male	148 (54.6%)
Female	128 (46.4%)
Medical history	
Hypertension	184 (70.2%)
Diabetes	56 (21.0%)
Hyperlipidemia	49 (18.7%)
History of stroke	43 (21.6%)
Atrial fibrillation	42 (16.0%)
Coronary artery disease	31 (11.8%)
Thyroid disease	21 (8.0%)
Chronic obstructive pulmonary disease	21 (8.0%)
Congestive heart failure	20 (7.6%)
Chronic kidney disease	18 (6.9%)
Asthma	16 (6.1%)
Myocardial infarction	12 (4.6%)
Liver disease	8 (3.0%)
Deep vein thrombosis	8 (3.0%)
Obstructive sleep apnea	7 (2.7%)
Valve disease or surgery	7 (2.7%)
Social history	
Tobacco use	
Smoker	87 (52.1%)
Nonsmoker	80 (47.9%)
Current alcohol use	68 (39.5%)
Current marijuana use	10 (5.9%)

^1^ Note: frequencies were calculated based on patients with available data.

**Table 2:** Vitals, severity indicators at admission, and locations of hemorrhage ^1^.

Variable	Mean (SD) or n (%)
Vital signs	
Heart rate (beats/minute)	84.4 (18.9)
Systolic pressure (mmHg)	155.0 (35.2)
Diastolic pressure (mmHg)	82.7 (23.6)
Temperature (°C)	37.7 (1.0)
SpO_2_ (%)	97.1 (2.9)
Admission GCS	n = 275
8 or lower	83 (30.2%)
9 or higher	192 (69.8%)
Admission ICH Score	n = 250
Score 0	52 (20.8%)
Score 1	64 (25.6%)
Score 2	55 (22.0%)
Score 3	48 (19.2%)
Score 4	20 (8.0%)
Score 5	11 (4.4%)
Primary location of hemorrhage	n = 276
Lobar	111 (40.2%)
Deep	84 (30.4%)
Brainstem and cerebellum	30 (10.9%)
Uncertain	51 (18.5%)
Surgical interventions	n = 276
Yes	68 (24.6%)
No	208 (75.4%)

^1^ Note: frequencies were calculated based on patients with available data.

**Table 3:** Outcome assessments at discharge ^1^.

Variable	Mean (SD) or n (%)
Length of hospital stay (days)	8.7 (8.5)
In-hospital deaths	61 (22.1%)
Discharge GCS	n = 271
8 or lower	80 (29.5%)
9 or higher	191 (70.5%)
Discharge mRS	n = 209
Score 0 (no symptoms)	12 (5.7%)
Score 1 (no significant disability despite symptoms)	21 (10.1%)
Score 2 (slight disability)	23 (11.0%)
Score 3 (moderate disability)	18 (8.6%)
Score 4 (moderate severe disability)	42 (20.1%)
Score 5 (severe disability; requiring constant care)	32 (15.3%)
Score 6 (deceased)	61 (29.2%)
Discharge destination outcome classification	n = 276
Positive	80 (29.0%)
Negative	135 (48.9%)
Death	61 (22.1%)

^1^ Note: frequencies were calculated based on patients with available data.

**Table 4:** Spearman’s Correlation results between levels of bilirubin or albumin and variables of stroke severity or outcomes (n = 276) ^1^.

r_s_ *p*-Value (n) ^1^	Total Bilirubin	Direct Bilirubin	Indirect Bilirubin	Albumin
Admission GCS	−0.08 0.1978 (275)	−0.17 0.0114 * (225)	−0.03 0.6722 (225)	0.13 0.0269 * (271)
Admission ICH Score	0.07 0.2537 (250)	0.19 0.0075 ** (204)	0.03 0.6332 (204)	−0.06 0.3822 (247)
Discharge GCS	−0.07 0.2668 (271)	−0.12 0.0661 (222)	0.01 0.8940 (222)	0.15 0.0133 * (267)
Discharge mRS	0.09 0.1991 (209)	0.15 0.0454 * (171)	0.06 0.4146 (171)	−0.16 0.0227 * (207)
Length of hospital stay	−0.09 0.1553 (276)	0.01 0.8882 (226)	−0.07 0.3274 (226)	−0.03 0.5774 (272)

^1^ Each analysis included only patients with available data, thus explaining the different numbers of patients analyzed in each cell. * *p* < 0.05, ** *p* < 0.01.

## Data Availability

The data used to support the findings of this study are available from the corresponding author upon request.

## Supplementary Materials

The following are available online at https://www.mdpi.com/article/10.3390/antiox10091346/s1, Table S1: Exclusion criteria for subject selection; Table S2: Lab measurements of specimen collected within 7 days after admission; Table S3: Correlations between levels of bilirubin (total, direct, and indirect) and albumin with age, vital signs, and other lab measurements for all patients (n = 276); Table S4: Wilcoxon rank sum or Spearman’s correlation test results between variables of interest and discharge mRS.
