# Supplementary material for: Association of Serum Bilirubin with the Severity and Outcomes of Intracerebral Hemorrhages
Source: Antioxidants (Basel). 2021 Aug 25;10(9):1346. doi: 10.3390/antiox10091346 (PMC8465680; doi:10.3390/antiox10091346)
Supplement: Supplementary file 1 [file antioxidants-10-01346-s001.zip › antioxidants-1323600-supplementary.pdf]

Supplementary Materials

Table S1: Exclusion criteria for subject selection.

Exclusion Criteria	Diagnosis or Identification Codes
Pregnancy	ICD-9: V22; ICD-10: Z33
Cocaine abuse or dependence	ICD-9: 304.2, 305.6; ICD-10: F14.1, F14.2
Amphetamine abuse or dependence	ICD-9: 304.4, 305.7; ICD-10: F15.1, F15.2
Sepsis	ICD-9: 995.91, 995.92; ICD-10: A02.1, A32.7, A40, A41, A54.86, B37.7, R65.2
Medications	
Irinotecan	RXNORM: 51499, 153329, 1719771
Tolbutamide	RXNORM: 10635, 374152
Ampicillin	RXNORM: 21615, 376673, 1659591, 37415
Ceftriaxone	RXNORM: 2193, 692977, 605946
Pazopanib	RXNORM: 714438
Amitriptyline	RXNORM: 704, 214220, 282427
Desipramine	RXNORM: 3247
Imipramine	RXNORM: 5691
Chlordiazepoxide	RXNORM: 214220, 2356, 611854, 606640
Diazepam	RXNORM: 3322

Table S2: Lab measurements of specimen collected within 7 days after admission.

Lab Measurements	Mean (Median)
Direct bilirubin (mg/dL)	0.16 (0.10)
Indirect bilirubin (mg/dL)	0.51 (0.35)
Total bilirubin (mg/dL)	0.65 (0.45)
Albumin (g/dL)	3.72 (3.80)
Platelet (thou/mm^3)	208.74 (207.00)
Hematocrit (%)	39.64 (39.40)
Hemoglobin (g/dL)	13.12 (13.20)
WBC (thou/mm^3)	10.75 (9.70)
HDL (mg/dL)	55.56 (52.00)
LDL (mg/dL)	88.50 (83.00)
ALT (IU/L)	24.67 (19.00)
AST (IU/L)	33.76 (26.00)
Alkaline phosphatase (IU/L)	80.54 (72.00)
Glucose (mg/dL)	149.51 (136.00)
Hemoglobin A1c (%)	5.97 (5.70)
Lactate (mmol/L)	2.14 (1.50)
INR	1.20 (1.10)
PT (seconds)	14.76 (13.90)
aPTT (seconds)	30.87 (29.00)
TSH (mIU/L)	2.09 (1.56)
Free T4 (ng/dL)	1.19 (1.19)
Vitamin B12 (pg/mL)	634.62 (542.00)
Folate (ng/mL)	17.57 (19.80)

Abbreviations: WBC, White Blood Cell; HDL, High-Density Lipoprotein; LDL, Low-Density Lipoprotein; ALT, Alanine Aminotransferase; AST, Aspartate Aminotransferase; INR, International Normalized Ratio; PT, Prothrombin Time; aPTT, Activated Partial Thromboplastin Time; TSH, Thyroid Stimulating Hormone; Free T4, Free Thyroxine.

Table S3: Correlations between levels of bilirubin (total, direct, and indirect) and albumin with age, vital signs, and other lab measurements for all patients (n=276).

r_s<br>p-value<br>(n)[(1)]	Total bilirubin	Direct bilirubin	Indirect bilirubin	Albumin
Age	0.04<br>0.5513<br>(276)	-0.06<br>0.3619<br>(226)	0.15<br>0.0289*<br>(226)	0.04<br>0.5055<br>(272)
Systolic blood pressure	-0.02<br>0.7082<br>(275)	-0.14<br>0.0383*<br>(225)	-0.01<br>0.8943<br>(225)	0.21<br>0.0006**<br>(271)
Diastolic blood pressure	-0.13<br>0.0336*<br>(275)	-0.15<br>0.0262*<br>(225)	-0.15<br>0.0253*<br>(225)	0.19<br>0.0016**<br>(271)
Hemoglobin	0.11<br>0.0739<br>(276)	-0.05<br>0.4661<br>(226)	0.16<br>0.0182*<br>(226)	0.25<br><.0001***<br>(272)
Hematocrit	0.07<br>0.2292<br>(275)	-0.07<br>0.3219<br>(225)	0.11<br>0.0925<br>(225)	0.24<br><.0001***<br>(271)
WBC	-0.02<br>0.7183<br>(275)	-0.03<br>0.6084<br>(226)	-0.06<br>0.3749<br>(226)	-0.00<br>0.9744<br>(271)
Platelets	-0.32<br><.0001***<br>(275)	-0.27<br><.0001***<br>(226)	-0.27<br><.0001***<br>(226)	0.13<br>0.0314*<br>(271)
Glucose	-0.03<br>0.6157<br>(273)	-0.02<br>0.7785<br>(223)	-0.08<br>0.2479<br>(223)	0.06<br>0.3055<br>(269)
LDL	-0.08<br>0.3140<br>(147)	-0.18<br>0.0475*<br>(123)	-0.05<br>0.5821<br>(123)	0.06<br>0.4821<br>(144)
HDL	-0.12<br>0.1383<br>(146)	-0.04<br>0.6847<br>(122)	-0.15<br>0.0941<br>(122)	0.19<br>0.0247*<br>(143)
ALT	0.17<br>0.0045**<br>(272)	0.14<br>0.0400*<br>(222)	0.15<br>0.0289*<br>(222)	0.04<br>0.5103<br>(271)
AST	0.27<br><.0001***<br>(272)	0.22<br>0.0010**<br>(222)	0.28<br><.0001***<br>(222)	0.02<br>0.7551<br>(271)

*p<0.05, **p<0.01, ***p<0.001.

[(1)] Note: our analysis included only patients with available data, thus explaining the different numbers of patients analyzed in each cell. WBC=White Blood Cell, HDL=High-Density Lipoprotein, LDL=Low-Density Lipoprotein, ALT=Alanine Aminotransferase, AST=Aspartate Aminotransferase.

Table S4. Wilcoxon rank sum or Spearman's correlation test results between variables of interest and discharge mRS.

Variable	Discharge mRS Median (IQR)	r_s	*p*-value
Age		-0.09	0.176
Sex			0.852
Male	4.0 (3.0)		
Female	4.0 (4.0)		
Heart rate at admission		0.18	0.011*
Systolic pressure at admission		0.00	0.994
Diastolic pressure at admission		0.05	0.494
Temperature at admission		-0.22	0.002**
SpO_2 at admission		0.19	0.008**
Alcohol use			0.298
Yes	4.0 (4.0)		
No	4.0 (3.0)		
Smoking history			0.046*
Never smoker	3.0 (2.5)		
Former smoker	4.0 (3.0)		
Current smoker	4.0 (3.0)		
Atrial fibrillation			0.222
Yes	4.0 (3.0)		
No	4.0 (4.0)		
Coronary artery disease			0.245
Yes	4.0 (3.0)		
No	4.0 (4.0)		
Chronic obstructive pulmonary disease			
Yes	5.0 (3.0)		0.832
No	4.0 (4.0)		
Chronic heart failure			
Yes	5.0 (3.5)		0.433
No	4.0 (4.0)		
Diabetes			0.436
Yes	4.0 (3.0)		
No	4.0 (4.0)		
Myocardial infarction			
Yes	4.0 (3.0)		0.433
No	4.0 (4.0)		
Dyslipidemia			
Yes	4.0 (3.0)		0.212
No	4.0 (4.0)		
History of stroke			0.180
Yes	4.0 (3.5)		
No	4.0 (4.0)		
Obstructive sleep apnea			
Yes	4.0 (2.0)		0.593
No	4.0 (4.0)		
Liver disease			
Yes	4.0 (3.0)		0.719
No	4.0 (4.0)		

Chronic kidney disease			
Yes	4.0 (4.0)		0.697
No	4.0 (4.0)		
Valve disease or surgery			
Yes	5.5 (1.0)		0.075
No	4.0 (4.0)		
Primary location of hemorrhage			
Deep	4.0 (3.0)		<0.001***
Lobar	4.0 (3.0)		
Brainstem and cerebellum	4.0 (3.0)		
Unknown	6.0 (2.0)		
Surgical intervention			0.070
Yes	5.0 (1.0)		
No	4.0 (4.0)		
Laboratory measurements			
Total bilirubin		0.09	0.188
Direct bilirubin		0.16	0.038*
Indirect bilirubin		0.07	0.384
Albumin		-0.16	0.023*
LDL		-0.18	0.042*
WBC		0.32	<0.001***
Glucose		0.38	<0.001***
AST		0.26	<0.001***
Lactate		0.24	0.011*
Troponin T		0.17	0.043*

*p<0.05, **p<0.01, ***p<0.001.
